# Risk prediction model for cervical lymph node metastasis of papillary thyroid microcarcinoma: a systematic review and meta-analysis

**DOI:** 10.3389/fendo.2025.1709773

**Published:** 2025-11-18

**Authors:** Xiaoli He, Qiang Zhang, Kaiju Yang, Jian Li, Mingzhu Luo, Ruihan Liu, Xi Yang

**Affiliations:** 1 Department of Ultrasound Medicine, Shapingba Hospital, Chongqing University (Shapingba District People’s Hospital, Chongqing), Chongqing, China; 2 Department of Scientific Research, Shapingba Hospital, Chongqing University (Shapingba District People’s Hospital, Chongqing), Chongqing, China

**Keywords:** papillary thyroid microcarcinoma, lymph node metastasis, prediction model, systematic review, meta-analysis

## Abstract

**Background:**

A growing number of risk prediction models for cervical lymph node metastasis (CLNM) in papillary thyroid microcarcinoma (PTMC) have been developed, but their performance and methodological rigor remain unclear. This study systematically reviews these models to evaluate their predictive performance and critically appraise their risk of bias.

**Methods:**

We conducted a systematic search of seven databases up to July 29, 2025. The methodological quality of the included studies was assessed using PROBAST. Model performance, measured by the area under the curve (AUC), was pooled using a random-effects meta-analysis.

**Results:**

A total of 15 studies, comprising 24 predictive models, were included. The pooled AUC was 0.794 (95% CI: 0.769–0.820), but with substantial heterogeneity (*I*^2^ = 89.6%). Subgroup analysis revealed a performance drop from the training set (pooled AUC, 0.812) to the validation set (pooled AUC, 0.774). The PROBAST assessment revealed that 12 of the 15 studies (80%) were critically at a high risk of bias, primarily due to flaws in participant selection.

**Conclusion:**

Although existing CLNM prediction models for PTMC show moderate to good discrimination on average, their clinical utility is severely limited by widespread methodological weaknesses and a high risk of bias. The current evidence is not robust enough to recommend any specific model for routine clinical use, and future research must prioritize methodological rigor and independent external validation.

## Introduction

Papillary thyroid carcinoma (PTC) is the most common type of thyroid cancer (1, 2), accounting for approximately 85% to 90% of all thyroid malignancies (3, 4). In recent years, the incidence of PTC has been steadily increasing, making it one of the fastest-growing malignant tumors worldwide (5–9). A significant portion of these diagnoses are for papillary thyroid microcarcinoma (PTMC), defined as PTC with a diameter of not more than 10 mm (10). Although PTMC is often considered a low-risk disease, it has a notable tendency to metastasize to cervical lymph nodes relatively early (11), with reported metastasis rates ranging from 30% to 82% (12–16). This creates a significant clinical dilemma, as the presence of cervical lymph node metastasis (CLNM) is a key factor in determining the appropriate management strategy, which can range from active surveillance to surgical intervention (17–22).

To address this clinical uncertainty, risk prediction models have emerged as a promising tool to help stratify patients and guide treatment decisions. The traditional detection of CLNM primarily relies on ultrasound examination, but this modality has relatively low sensitivity in identifying metastatic lymph nodes (23–25). Consequently, there is an urgent need to identify CLNM risk more accurately to inform surgical planning (26). In response, a growing number of studies have developed multivariable prediction models, often presented as nomograms, that combine clinical, pathological, and imaging features to estimate the probability of CLNM in patients with PTMC.

Despite the proliferation of these models, their performance, reliability, and ultimate clinical utility have not been systematically and critically appraised. Many prediction model studies are known to suffer from methodological shortcomings, such as biased participant selection, inadequate handling of data, and insufficient validation, which can lead to overly optimistic performance estimates. The adoption of a poorly developed or validated model into clinical practice could lead to incorrect patient stratification, resulting in either unnecessary overtreatment or dangerous undertreatment. A comprehensive evaluation of the existing evidence is therefore essential.

Therefore, this study was conducted to systematically review existing prediction models for CLNM in PTMC, evaluate their predictive performance through meta-analysis, and critically appraise their methodological quality using Prediction model Risk Of Bias ASsessment Tool (PROBAST). The goal is to summarize the current evidence, highlight methodological limitations in the field, and provide guidance for both clinical practice and the development of future, more robust prediction models.

## Methods

This systematic review and meta-analysis was conducted and reported in accordance with the Preferred Reporting Items for Systematic Reviews and Meta-Analyses (PRISMA) 2020 statement.

### Literature search

We searched the Chinese Biomedical Literature Database, China National Knowledge Infrastructure (CNKI), Wanfang, PubMed, EMbase, Web of Science, and Cochrane Library databases to collect studies on the risk prediction model of CLNM in PTMC published from the establishment of the databases to July 29, 2025. We combined keywords with related free words. The search terms included thyroid microcarcinoma, ultrasound, radiomic, lymph node, transfer, nomogram, etc. Detailed search strategies for each database, including the specific combinations of medical subject headings (MeSH), Emtree terms, and free-text words, are provided in **Supplementary Appendix 1**.

### Inclusion and exclusion criteria

Based on the PICOTS framework, the inclusion and exclusion criteria for this study were as follows:

P (population): Patients with a postoperative pathological diagnosis of PTMC (defined as papillary thyroid carcinoma with tumor diameter ≤ 10 mm).

I (intervention model): Studies developing and/or validating a multivariable risk prediction model for CLNM.

C (comparator): Not applicable for this type of review.

O (outcome): Model performance metrics, primarily the area under the receiver operating characteristic curve (AUC).

T (timing): At the time of initial diagnosis, prior to surgical intervention.

S (setting): Eligible studies included observational study designs (e.g., retrospective or prospective cohort studies, case–control studies) that reported on the development or validation of a prediction model.

According to the qualification criteria, the included studies must be original research involving the CLNM risk prediction model of PTMC (excluding reports, reviews, conference papers, and meta-analyses), including both Chinese and English literature, with a focus on the training set and validation set of the prediction model. The exclusion criteria are studies with only training set but no validation set and studies with incomplete data or where the original text cannot be obtained.

### Literature screening and data extraction

Two researchers independently conducted a preliminary screening of the titles and abstracts of the articles based on preset inclusion and exclusion criteria. Subsequently, the full-text reading of the literature that passed the initial screening was conducted to further screen whether it met the inclusion criteria. Data extraction uses standardized tables based on CHARMS (Checklist for Critical Appraisal and Data Extraction for Systematic Reviews of Prediction Modelling Studies) checklist. The extracted data were as follows: author, publication year, country, sample source, sample size, study type, age, lymph node metastasis rate, diagnostic basis, modeling sample size, model performance, model validation, main predictors, clinical applicability assessment, and lymph node metastasis risk stratification. During the process of article screening and data extraction, if there are any differences, a third researcher was introduced for discussion.

### Risk assessment of bias

The risk of bias and suitability of the included studies were evaluated using the PROBAST checklist (27, 28). Two researchers independently assessed the risk of bias and applicability. The PROBAST checklist contains 20 signaling questions across four evaluation domains: participants, predictors, results, and analysis. The answer options for each signaling question are “yes”, “probably yes”, “no”, “probably no”, or “no information”. If the answer to any question in a certain field is “no” or “probably no”, that field is judged to have a high risk of bias. Only when all domains are determined to have a low risk of bias can the overall risk of bias be recognized as low.

### Data synthesis and statistical analysis

Meta-analysis of the area under the curve (AUC) and 95% confidence interval (CI) of the model was conducted using Stata 14. Heterogeneity among studies was evaluated by using *Q* test and *I*^2^. If *P >*0.1 and *I*^2^ <50%, it is considered that the heterogeneity among studies is relatively small, and a fixed-effect model is selected. If *P* ≤0.1 and *I*^2^ ≥50%, the random-effects model is selected. Subgroup analysis was conducted based on the training set, validation set, and sources of predictors. Sensitivity analysis was performed using the elimination method one by one. Egger’s test was used to assess publication bias.

## Result

**Figure 1:**
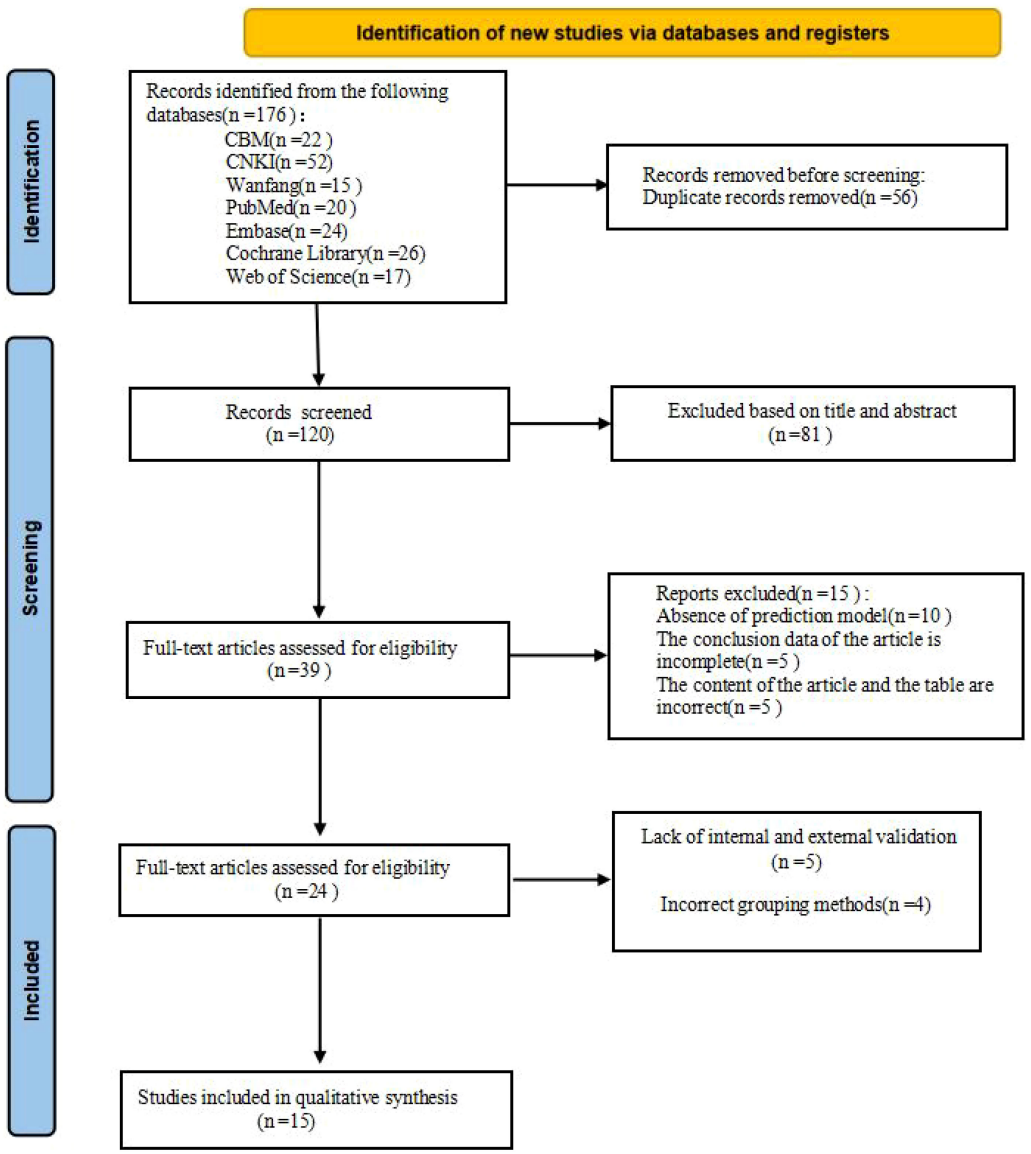
Flowchart of searching and screening steps for the studies.

A total of 176 studies were identified through database retrieval. After removing 56 duplicate records, the titles and abstracts of the remaining studies were screened, and 81 studies were excluded. Subsequently, full-text evaluations were conducted on 39 articles. During this process, 15 studies were excluded due to the lack of a predictive model or incomplete data. Nine studies were excluded due to the lack of internal and external validation or improper grouping methods. Ultimately, 15 studies (1, 2, 4–6, 10, 11, 14, 15, 17, 18, 22, 23, 25, 29) and 24 models were included in this meta-analysis (**Figure 1**).

### Basic characteristics included in the study

This meta-analysis included 15 retrospective studies published between 2021 and 2025, involving a total of 24 predictive models. As detailed in **Table 1**, the studies showed significant geographical diversity, with seven conducted in China and eight in Western countries. The total sample sizes varied considerably, ranging from 133 to 2,368 participants.

**Table 1:** Characteristics of the included studies.

Author, year	Country	Period	Sample source	Study type	Sample size	Total sample size
Ding et al., 2024 (10)	China	December 2020 to December 2021	Postoperative patients	Retrospective study	TS (*n* = 282), VS (*n* = 122)	404
He 2024 (1)	China	January 2016 to January2020	SEER database Postoperative patients	Retrospective study	TS (*n* = 2085), VS (*n* = 283)	2,368
Jin et al., 2025 (11)	China	January 2022 to April 2023	Postoperative patients	Retrospective study	TS (*n* = 92), VS (*n* = 41)	133
Luo 2021 (3)	China	October 2014 to October 2020	Postoperative patients	Retrospective study	TS (*n* = 382), VS (*n* = 164)	546
Yu 2024 (4)	China	April 2022 to April 2023	Postoperative patients	Retrospective study	TS (*n* = 128), VS (*n* = 56)	184
Huang et al., 2021 (17)	Switzerland	January 2018 to February 2020	Institution Database	Retrospective study	TS (*n* = 439), VS (*n* = 220)	659
Zhu et al., 2023 (5)	USA	February 2016 to June 2022	Postoperative patients	Retrospective study	TS (*n* = 151), VS (*n* = 65)	216
Duan et al., 2024 (14)	China	January 2017 to December 2022	Postoperative patients in 2 hospitals	Retrospective study	TS (*n* = 709), VS (*n* = 302)	1,011
Gao et al., 2025 (29)	USA	October 2020 to October 2022	Postoperative patients in 2 hospitals	Retrospective study	TS (*n* = 201), VS (*n* = 87)	288
Zhang et al., 2021 (23)	Switzerland	January 2018 to December 2020	Postoperative patients	Retrospective study	TS (*n* = 180), VS (*n* = 89)	269
Liu et al., 2024 (6)	UK	January 2018 to December 2020	Postoperative patients in 2 hospitals	Retrospective study	TS (*n* = 327), VS (*n* = 153)	460
Qiu et al., 2024 (2)	UK	July 2019 to December 2021	Postoperative patients	Retrospective study	TS (*n* = 269), VS (*n* = 108)	377
Wu et al., 2024 (18)	Switzerland	November 2021 to October 2022	Postoperative patients	Retrospective study	TS (*n* = 142), VS (*n* = 62)	204
Zhang et al., 2024 (25)	Romania	June 2020 to May 2021	Postoperative patients	Retrospective study	TS (*n* = 445), VS (*n* = 191)	636
Zhao et al., 2023 (15)	China	January 2020 to July 2022	Postoperative patients	Retrospective study	TS (*n* = 214), VS (*n* = 55)	369

TS, training set; VS, validation set.

Further details on participant demographics are summarized in **Table 2**. The mean age of the patient cohorts was consistent across studies, ranging from 43 to 48 years. The reported rate of cervical lymph node metastasis (CLNM) was substantial, falling between 30% and 50%. A critically key methodological point highlighted in **Table 2** is that only three studies explicitly confirmed that their population met the strict definition of PTMC (tumor diameter ≤10 mm), and quality control for ultrasound imaging was not reported in four of the studies.

**Table 2:** Other characteristics of the included studies.

Author, year	Gender (male/female)	Mean age, years	Lymph node partitioning	CLNM (%)	PTMC ≤10 mm	Image quality control
Ding et al., 2024	120/284	45.20	Neck	159 (31.5)	–	–
He 2024	392/1976	46.50	Neck	878 (37.1)	–	–
Jin et al., 2025	30/103	47.63	Neck	57 (42.8)	–	Two doctors blind
Luo 2021	126/420	47.00	Neck	163 (29.9)	–	–
Yu 2024	37/147	44.51	Neck	81 (44.0)	–	One extraction, one review
Huang et al., 2021 (17)	152/507	43.15	Neck	237 (35.9)	–	Third calibration
Zhu et al., 2023 (5)	42/174	43.62	Neck	102 (47.2)	+	Third calibration
Duan et al., 2024 (14)	775/254	46.5	Neck	465 (45.1)	–	Third calibration
Gao et al., 2025 (29)	66/222	48.25	Neck	95 (32.9)	–	Two doctors independently extracted
Zhang et al., 2021 (23)	775/254	46.5	Neck	104 (38.6)	+	Two doctors independently extracted, controversial discussion
Liu et al., 2024 (6)	66/222	48.25	Neck	110 (40.8)	–	Two doctors independently extracted
Qiu et al., 2024 (2)	69/200	44.82	Neck	119 (31.5)	–	Two doctors independently extracted
Wu et al., 2024 (18)	67/393	45.6	Neck	102 (50)	–	Third calibration
Zhang et al., 2024 (25)	82/295	46.5	Neck	203 (31.9)	–	Two doctors independently extracted, controversial discussion
Zhao et al., 2023 (15)	58/311	43.89	Neck	184 (49.9)	+	–

+, mentioned; -, not mentioned.

A detailed overview of the 24 prediction models is presented in **Table 3**. The performance of these models, as measured by the area under the curve (AUC), varied widely, with validation set AUCs ranging from a modest 0.661 to a highly discriminative 0.921. The predictors used to build these models were diverse but frequently included age, gender, tumor size, capsular invasion, and microcalcification. As shown in **Table 3**, while most models were presented as nomograms, their methodological follow-through varied. Although 14 studies reported some form of model calibration, the method was often unspecified. Furthermore, while 12 studies assessed clinical utility using decision curve analysis, only four attempted to define risk strata for clinical application, highlighting a common gap between model development and practical implementation.

**Table 3:** Overview of the information of the included prediction models.

Author, year	Number of models	Calibration	Verification method	Model performance	Predictor	Model display	Clinical applicability assessment	Risk stratification
Ding et al., 2024	1	Hosmer–Lemeshow test	Interior	TS: 0.747 (95% CI: 0.690–0.804) VS: 0.778 (95% CI: 0.697–0.860)	Margin, size, multifocality, capsular invasion, recovery mode	Nomogram	Decision curve analysis	+
He 2024	1	Hosmer–Lemeshow test Calibration of 1,000 bootstrap samples	External	TS: 0.763 (95% CI: 0.728–0.799) VS: 0.725 (95% CI: 0.613–0.837)	Male, size (>5 mm), multifocality, capsular invasion	Nomogram	Decision curve analysis	–
Jin et al., 2025	3	There is calibration, but no method specified	Interior	Clinical prediction model: TS: 0.810 (95% CI: 0.673–0.948) VS: 0.769 (95% CI: 0.670–0.868) Radiomics scoring model: TS: 0.900 (95% CI: 0.840–0.961) VS: 0.860 (95% CI: 0.743–0.977) Conjunctive model: TS: 0.911 (95% CI: 0.855–0.96) VS: 0.903 (95% CI: 0.804–0.980)	Microcalcification, capsular invasion (≥25%), lymph node condition	Nomogram	Decision curve analysis	–
Luo 2021	1	Hosmer–Lemeshow test	Interior	TS: 0.775 (95% CI: 0.723–0.826) VS: 0.720 (95% CI: 0.635–0.804)	Age, gender, size, capsular invasion, multifocality, Hashimoto’s thyroiditis	Nomogram	Decision curve analysis	+
Yu 2024	2	Without calibration	Interior	Radiomics group: TS: 0.78 (95% CI: 0.74–0.82) VS: 0.72 (95% CI: 0.68–0.75) Joint group: TS: 0.87 (95% CI: 0.83–0.90) VS: 0.81 (95% CI: 0.78–0.83)	Capsular invasion, age	Nomogram	–	–
Huang et al., 2021 (17)	1	Hosmer–Lemeshow test	Interior	TS: 0.78 (95% CI: 0.735–0.825) VS: 0.77 (95% CI: 0.703–0.837)	Age, size, multifocality, capsular invasion	Nomogram	Decision curve analysis Clinical Impact Curve	–
Zhu et al., 2023 (5)	3	Hosmer–Lemeshow test	Interior	TS: 0.78 (95% CI: 0.735–0.825) VS: 0.77 (95% CI: 0.703–0.838)	BRAF V600E, age (<45), size (>5 mm), capsular invasion, microcalcification	Nomogram	Decision curve analysis	–
Duan et al., 2024 (14)	1	Bootstrapping	Interior External	TS: 0.784 (95% CI: 0.750–0.81) VS: 0.779 (95% CI: 0.729–0.830)	Age, multifocality, size, microcalcification, aspect ratio >1, lymph node condition, FT4, TPOAb	Nomogram	Decision curve analysis	+
Gao et al., 2025 (29)	3	Hosmer–Lemeshow test	Interior	Combined model: TS: 0.921 (95% CI: 0.883–0.958) VS: 0.889 (95% CI: 0.820–0.959)USA—Clinic: TS: 0.812 (95% CI: 0.748–0.876) VS: 0.741 (95% CI: 0.627–0.856) Imaging group score: TS: 0.876 (95% CI: 0.826–0.926) VS: 0.807 (95% CI: 0.713–0.901)	Age, capsular invasion, male, microcalcification	Nomogram	Decision curve analysis	–
Zhang et al., 2021 (23)	1	There is calibration, but no method specified	Interior	TS: 0.777 (95% CI: 708–0.847) VS: 0.661 (95% CI: 544–0.778)	Age, gender, size, capsular invasion, lymph node condition	Nomogram	–	–
Liu et al., 2024 (6)	1	There is calibration, but no method specified	External	TS: 0.795 (95% CI: 0.745–0.846) VS: 0.774 (95% CI: 0.696–0.852)	Rad grade, age, capsular invasion	Nomogram	–	–
Qiu et al., 2024 (2)	1	There is calibration, but no method specified	Interior	TS: 0.703 (95% CI: 0.640–0.731) VS: 0.672 (95% CI: 0.656–0.707)	Age, gender, size, multifocality, margin	Nomogram	Decision curve analysis	–
Wu et al., 2024 (18)	3	There is calibration, but no method specified	Interior	Clinic + US: TS: 0.835 (95% CI: 0.768–0.902) VS: 0.806 (95% CI: 0.699–0.914)Radiological group characteristics: TS: 0.734 (95% CI: 0.653–0.815) VS: 0.686 (95% CI: 0.547–0.824) Aggregative model: TS: 0.868 (95% CI: 0.811–0.925) VS: 0.857 (95% CI: 0.759–0.955)	Male, echo, margin, microcalcification	Nomogram	Decision curve analysis	–
Zhang et al., 2024 (25)	1	Hosmer–Lemeshow test	Interior	TS: 0.720 (95% CI: 0.649–0.791) VS: 0.704 (95% CI: 0.622–0.786)	Age, gender, size, capsular invasion, lymph node condition	Nomogram	Decision curve analysis	–
Zhao et al., 2023 (15)	1	modeFRONTIER	Interior	TS: 0.946 (95% CI: 0.920–0.972) VS: 0.845 (95% CI: 0.714–0.976)	Gender, age	Nomogram	Decision curve analysis	+

TS, training set; VS, validation set; +, something has been done; -, nothing has been done.

### Quality assessment results

The methodological quality of the 15 included studies was assessed using PROBAST. A detailed, item-by-item evaluation of the risk of bias and applicability concerns across the four key domains for each study is presented in **Table 4**.

**Table 4:** Prediction model risk of bias assessment tool evaluation results included in the study.

Study	Study type	ROB				Applicability			Overall	
		Participants	Predictors	Outcome	Analysis	Participants	Predictors	Outcome	ROB	Applicability
Ding et al., 2024	B	–	+	+	+	–	+	+	–	–
He 2024	B	–	+	+	+	–	+	+	–	–
Jin et al., 2025	B	–	+	+	+	–	+	+	–	–
Luo 2021	B	–	+	+	+	–	+	+	–	–
Yu 2024	B	–	+	+	–	–	+	+	–	–
Huang et al., 2021 (17)	B	–	+	+	+	–	+	+	–	–
Zhu et al., 2023 (5)	B	+	+	+	+	+	+	+	+	+
Duan et al., 2024 (14)	B	–	+	+	+	–	+	+	–	–
Gao et al., 2025 (29)	B	–	+	+	+	–	+	+	–	–
Zhang et al., 2021 (23)	B	+	+	+	+	+	+	+	+	+
Liu et al., 2024 (6)	B	–	+	+	+	–	+	+	–	–
Qiu et al., 2024 (2)	B	–	+	+	+	–	+	+	–	–
Wu et al., 2024 (18)	B	–	+	+	+	–	+	+	–	–
Zhang et al., 2024 (25)	B	–	+	+	+	–	+	+	–	–
Zhao et al., 2023 (15)	B	+	+	+	+	+	+	+	+	+

PROBAST, Prediction model Risk Of Bias ASsessment Tool; A, development only; B, development and validation in the same publication; +, low ROB/low concern regarding applicability; -, high ROB/high concern regarding application; ROB, risk of bias.

As detailed in **Table 4**, our analysis found that only three studies demonstrated an overall low risk of bias, while the remaining 12 were judged to have a high risk of bias. The specific domain responsible for the high risk of bias varied, namely: (1) Participant domain: This was the most common source of bias. A total of 12 studies were rated as having a high risk of bias in this domain, primarily due to the use of inappropriate inclusion criteria that did not strictly limit the tumor diameter to ≤10 mm; (2) Analysis domain: Only one study was rated as having a high risk of bias in this area. This was due to a failure to account for potential model overfitting or to assess the optimism of the model’s performance; (3) Predictors and outcome domains: All 15 studies were consistently rated as having a low risk of bias in these domains, indicating robust methods to define and measure predictors and outcomes.

In summary, the granular results in **Table 4** reveal that while predictor and outcome assessments were methodologically sound, critical issues in participant selection were the predominant reason for the high risk of bias observed in the majority of the included literature.

### Meta-analysis

**Figure 2:**
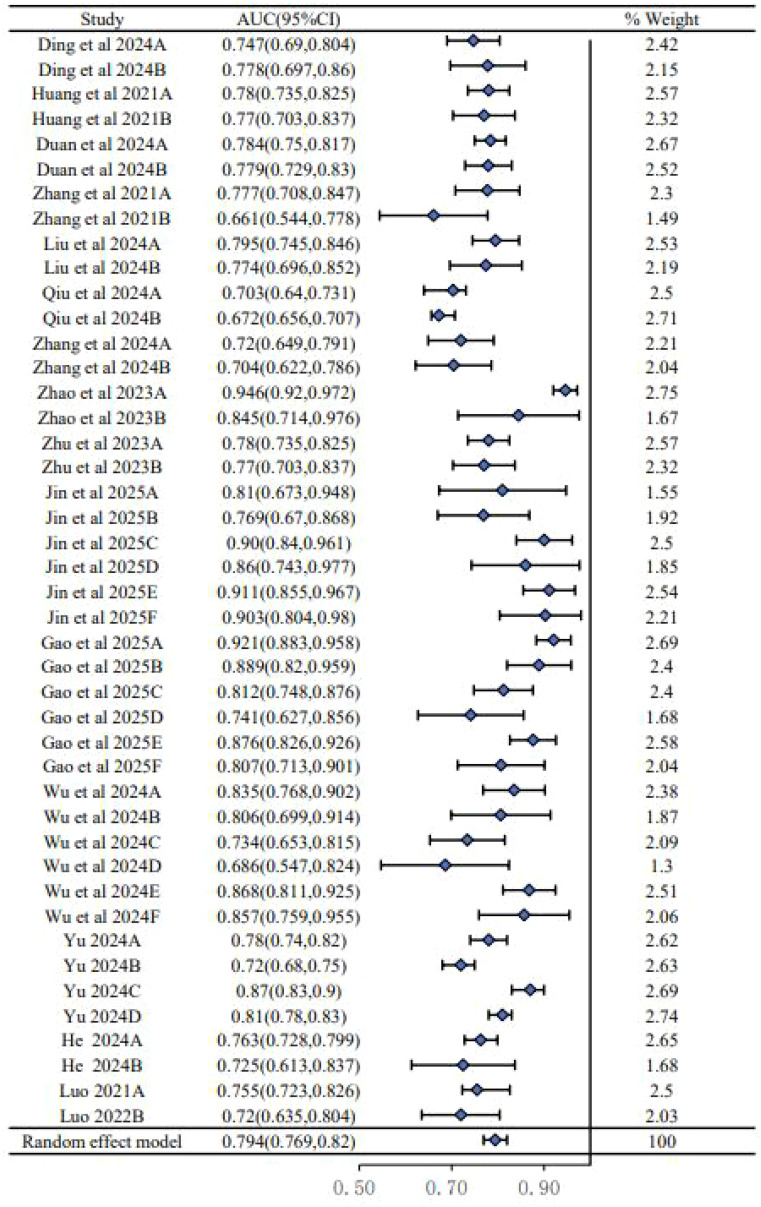
Forest plot showing the meta-analysis of the area under the curve for 15 studies.

We conducted a meta-analysis of the AUC values from 24 models reported in the 15 included studies. As shown in the forest plot (**Figure 2**), there was substantial heterogeneity among the studies (*I*^2^ = 89.6%, *P* < 0.00001), necessitating the use of a random-effects model. This high level of heterogeneity suggests a significant variability in model performance across different study populations, predictor definitions, and modeling techniques. The pooled AUC was 0.794 (95% CI: 0.769–0.820), indicating moderate to good overall discriminatory performance, though this result must be interpreted with caution given the aforementioned heterogeneity and high risk of bias in the primary studies.

### Subgroup analysis

**Figure 3:**
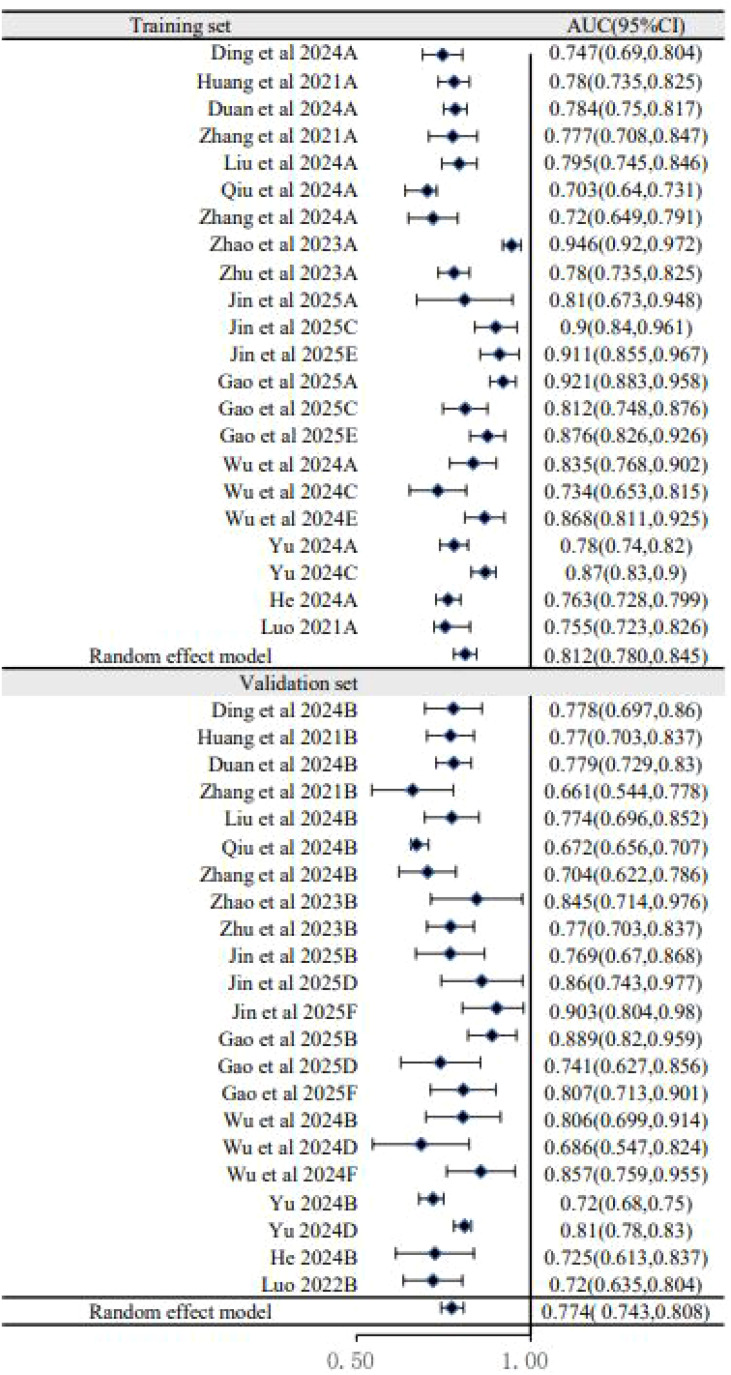
Subgroup analysis of forest plot (training versus validation).

**Figure 4:**
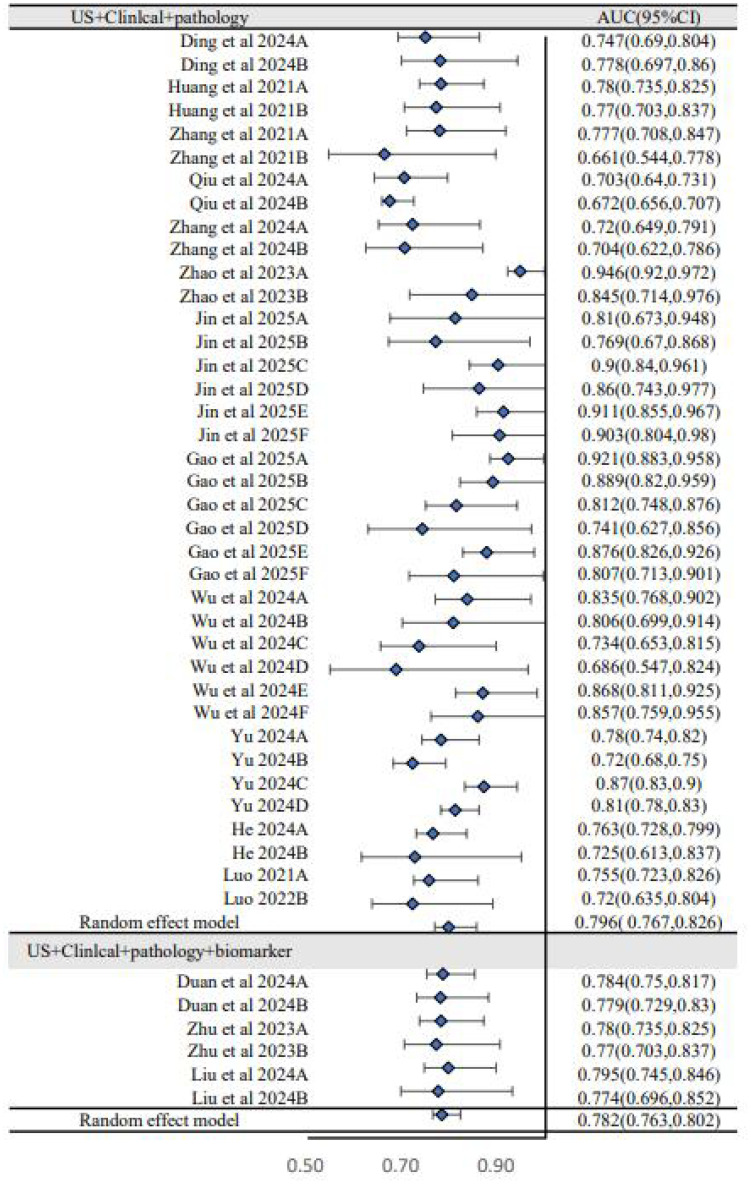
Subgroup analysis of forest plot (US+clinical+pathology versus US+clinical+pathology+biomarker).

To explore sources of heterogeneity, subgroup analyses were performed. The analysis based on dataset type (**Figure 3**) showed that the pooled AUC of models in the training sets was 0.812 (95% CI: 0.780–0.845), which was higher than the pooled AUC in the validation sets (0.774, 95% CI: 0.743–0.808). This performance drop-off, or “optimism,” is common in prediction model studies and underscores the critical importance of external validation to assess a model’s true generalizability. A second subgroup analysis based on predictor types (**Figure 4**) showed that models combining US, clinical, and pathology features had a pooled AUC of 0.796 (95% CI: 0.767–0.826), while the further addition of biomarkers did not improve performance (AUC = 0.782, 95% CI: 0.763–0.802). This suggests that the biomarkers included in current models may have limited incremental predictive value over more established factors.

### Sensitivity analysis

**Figure 5:**
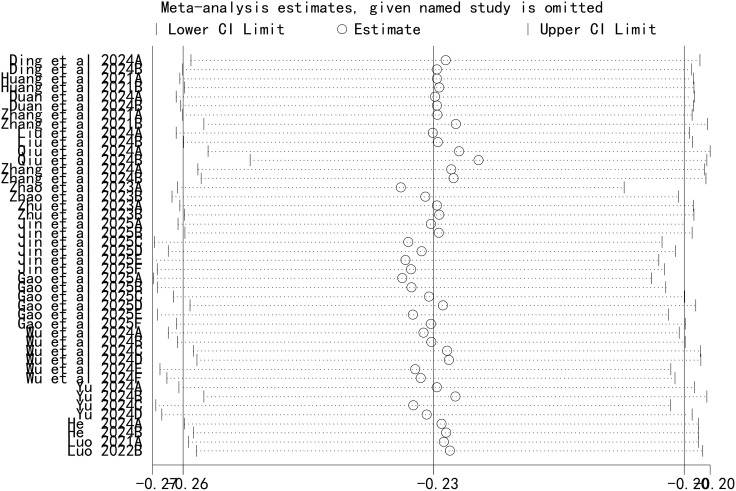
Sensitivity analysis.

A leave-one-out sensitivity analysis was performed to assess the stability of our findings, with the results shown in **Figure 5**. This analysis demonstrated that the sequential removal of each individual study did not significantly alter the overall pooled AUC. This confirms that our meta-analysis results are robust and not unduly influenced by any single study.

### Publication bias

Publication bias was evaluated using Egger’s test. The results showed that there was no significant publication bias in the included studies (*P* = 0.103).

## Discussion

This systematic review and meta-analysis synthesized evidence from 15 studies involving 24 prediction models for CLNM in patients with PTMC. The principal findings are threefold, namely: first, the models demonstrate moderate to good discriminatory ability on average, with a pooled AUC of 0.794; second, this overall performance estimate is subject to considerable statistical heterogeneity (*I*^2^ = 89.6%) across studies; and third and most critically, the validity of these findings is severely threatened by the methodological quality of the underlying evidence, as 12 of the 15 included studies were judged to be at a high risk of bias according to PROBAST.

The pooled AUC of 0.794 suggests that, on paper, these models can distinguish between patients with and without CLNM reasonably well. However, this figure likely represents an overestimation of true performance. The subgroup analysis revealed a consistent drop in performance from the training sets (pooled AUC 0.812) to the validation sets (pooled AUC 0.774), a phenomenon known as “optimism” that is characteristic of models that have not been robustly validated on independent data. This performance degradation, combined with the high risk of bias identified in most studies—particularly related to participant selection and inadequate handling of potential overfitting—means that the reported performance metrics should be interpreted with extreme caution.

A significant challenge in synthesizing these models is the inconsistent role and definition of certain predictors. In terms of predictors, US and clinicopathological features such as age, gender, tumor size, and capsular invasion are the most common inclusion indicators (30). However, the contribution of some biomarkers is contentious—for instance, TPOAb was identified as a protective factor in some studies (14, 31–33), potentially through an antibody-mediated cytotoxic effect. This contradicts other reports, such as those by Sun et al. (34) and Wang et al. (35), where TPOAb was associated with an increased risk of CLNM. These discrepancies may arise from differences in study populations (e.g., underlying rates of Hashimoto’s thyroiditis), variations in TPOAb detection assays, or failure to adjust for key confounders. The predictive value of the BRAF V600E mutation likewise remains debated. While some included studies (5) found it to be a strong predictor of metastasis, consistent with a large meta-analysis by Attia et al. (36), another research by Virk et al. (37) suggests that it is not a reliable predictor in the specific context of PTMC. This highlights the need for further research to clarify the role of molecular markers before they can be reliably incorporated into clinical prediction models.

The high risk of bias found in 80% of the included studies is a major concern that limits the clinical applicability of these models. As detailed in **Table 4**, the primary issue was in the participant domain, where many studies failed to strictly define PTMC (≤10 mm) or used retrospective designs with inadequate sampling strategies, which can introduce selection bias. Such methodological flaws not only inflate performance metrics but also render the models unreliable for use in the specific clinical population they are intended for. Furthermore, the lack of calibration assessment in many studies is a critical omission, as a model with good discrimination (AUC) can still be clinically useless if its predicted probabilities are poorly calibrated with observed frequencies.

The strengths of our review include a comprehensive search strategy across multiple databases, duplicate data extraction and quality assessment to minimize error, and a formal risk of bias assessment using the recommended PROBAST. However, this study also has its shortcomings. Firstly, all of the included studies were retrospective studies, and there was a certain degree of bias in the included population. Secondly, most of the studies were single-center studies, and some had relatively small sample sizes. Finally, some of the included studies did not strictly control the quality of ultrasound images, and some results might have been influenced by the experience of the operators and instruments used.

## Conclusion

In conclusion, while numerous CLNM risk prediction models for PTMC exist and demonstrate moderate discriminatory performance on average, their reliability and clinical utility are severely hampered by widespread methodological weaknesses and a high risk of bias. The current evidence base is not robust enough to recommend any single model for routine clinical use. Future research must prioritize methodological rigor, adhering strictly to development and reporting guidelines such as TRIPOD and PROBAST. Emphasis should be placed on large, multi-center prospective studies with independent external validation to develop truly reliable and well-calibrated models that can safely guide individualized treatment for patients with PTMC.

## Data Availability

The original contributions presented in the study are included in the article/**Supplementary Material**. Further inquiries can be directed to the corresponding authors.

## References

[1] Hualin H . To Develop and Validate a Nomogram Model for Predicting High Volume (>5) Central Lymph Node Metastasis in Papillary Thyroid Microcarcinoma. Hualin He: North China University of Science and Technology (2024). doi: 10.27108/d.cnki.ghelu.2024.001051. 42413301

[2] Qiu P Guo Q Pan K Lin J . Development of a nomogram for prediction of central lymph node metastasis of papillary thyroid microcarcinoma. BMC Cancer. (2024) 24:235. doi: 10.1186/s12885-024-12004-3, PMID: 38378515 PMC10877775

[3] Zhaoten L . Development and Validation of a Nomogram Predictive Model for Central Lymph Node Metastasis Risk in the Papillary Thyroid Microcarcinoma. Zhaoten Luo: University Of South China (2023). doi: 10.27234/d.cnki.gnhuu.2023.000555.

[4] Yixing Y . Gray-scale Ultrasound-based Radiomics for Prediction of Lymph Node Metastasis in Papillary Thyroid Microcarcinoma. Yixing Yu: Henan University (2024). doi: 10.27114/d.cnki.ghnau.2024.002830.

[5] Zhu D Wu X Zhang L Chen Z . Predictive value of ultrasound imaging characteristics and a BRAF V600E nomogram for central lymph node metastasis risk in papillary thyroid microcarcinoma. Altern Ther Health Med. (2023) 29:139–43., PMID: 37632946

[6] Liu J Yu J Wei Y Li W Lu J Chen Y . Ultrasound radiomics signature for predicting central lymph node metastasis in clinically node-negative papillary thyroid microcarcinoma. Thyroid Res. (2024) 17:4. doi: 10.1186/s13044-024-00191-x, PMID: 38369523 PMC10875890

[7] Cabanillas ME McFadden DG Durante C . Thyroid cancer. Lancet. (2016) 388:2783–95. doi: 10.1016/s0140-6736(16)30172-6, PMID: 27240885

[8] La Vecchia C Malvezzi M Bosetti C Garavello W Bertuccio P Levi F . Thyroid cancer mortality and incidence: a global overview. Int J Cancer. (2015) 136:2187–95. doi: 10.1002/ijc.29251, PMID: 25284703

[9] Möller M Gustafsson U Rasmussen F Persson G Thorell A . Natural course vs interventions to clear common bile duct stones: data from the Swedish Registry for Gallstone Surgery and Endoscopic Retrograde Cholangiopancreatography (GallRiks). JAMA Surg. (2014) 149:1008–13. doi: 10.1001/jamasurg.2014.249, PMID: 25133326

[10] Jiaojiao D Wei H Junxi G Tao S . Risk of cervical lymph node metastasis of thyroid micropapillary carcinoma predicted by constructing a nomogram based on contrast-enhanced ultrasound features. J Xinjiang Med University. (2024) 47:39–45 + 50. doi: 10.3969/j.issn.1009-5551.2024.01.007

[11] Bin J Feng C Lingling M Chenying L . Development and validation of an ultrasound radiomics-based nomogram for preoperative prediction of central lymph node metastasis in patients with papillary thyroid microcarcinoma. Zhejiang Medical. (2025) 47:1166–72. doi: 10.12056/j.issn.1006-2785.2025.47.11.2024-1739

[12] Gao X Luo W He L Cheng J Yang L . Predictors and a prediction model for central cervical lymph node metastasis in papillary thyroid carcinoma (cN0). Front Endocrinol (Lausanne). (2021) 12:789310. doi: 10.3389/fendo.2021.789310, PMID: 35154002 PMC8828537

[13] Shindo M Wu JC Park EE Tanzella F . The importance of central compartment elective lymph node excision in the staging and treatment of papillary thyroid cancer. Arch Otolaryngol Head Neck Surg. (2006) 132:650–4. doi: 10.1001/archotol.132.6.650, PMID: 16785411

[14] Duan S Yang Z Wei G Chen S Hu X Ryu YJ . Nomogram for predicting the risk of central lymph node metastasis in papillary thyroid microcarcinoma: a combination of sonographic findings and clinical factors. Gland Surg. (2024) 13:1016–30. doi: 10.21037/gs-24-154, PMID: 39015718 PMC11247594

[15] Zhao Y Fu J Liu Y Sun H Fu Q Zhang S . Prediction of central lymph node metastasis in patients with papillary thyroid microcarcinoma by gradient-boosting decision tree model based on ultrasound radiomics and clinical features. Gland Surg. (2023) 12:1722–34. doi: 10.21037/gs-23-456, PMID: 38229842 PMC10788563

[16] Zheng X Peng C Gao M Zhi J Hou X Zhao J . Risk factors for cervical lymph node metastasis in papillary thyroid microcarcinoma: a study of 1,587 patients. Cancer Biol Med. (2019) 16:121–30. doi: 10.20892/j.issn.2095-3941.2018.0125, PMID: 31119052 PMC6528461

[17] Huang C Cong S Shang S Wang M Zheng H Wu S . Web-based ultrasonic nomogram predicts preoperative central lymph node metastasis of cN0 papillary thyroid microcarcinoma. Front Endocrinol (Lausanne). (2021) 12:734900. doi: 10.3389/fendo.2021.734900, PMID: 34557165 PMC8453195

[18] Wu L Zhou Y Li L Ma W Deng H Ye X . Application of ultrasound elastography and radiomic for predicting central cervical lymph node metastasis in papillary thyroid microcarcinoma. Front Oncol. (2024) 14:1354288. doi: 10.3389/fonc.2024.1354288, PMID: 38800382 PMC11116610

[19] Hong C Tingyue Q . Application progress of contrast-enhanced ultrasonography in thyroid carcinoma diagnosis and treatment. J Ultrasound Clin Med. (2018) 20:478–80. doi: 10.16245/j.cnki.issn1008-6978.2018.07.013

[20] Zhan J Ding H . Application of contrast-enhanced ultrasound for evaluation of thyroid nodules. Ultrasonography. (2018) 37:288–97. doi: 10.14366/usg.18019, PMID: 30213158 PMC6177690

[21] Aifang B Jianlei Z Nini Z Yi Y Yan Q . The diagnostic value of ultrasonography for the nodular goiter combined with papillary thyroid microcarcinoma. Prog Modern Biol. (2015) 15:6538–41. doi: 10.13241/j.cnki.pmb.2015.33.037

[22] Qianwen L . Development and Validation of a Nomogram Predictive Model for Central Lymph Node Metastasis Risk in the Papillary Thyroid Microcarcinoma. Qianwen Luo: North China University of Science and Technology (2021). doi: 10.27108/d.cnki.ghelu.2021.000200.

[23] Zhang L Ling Y Zhao Y Li K Zhao J Kang H . A nomogram based on clinicopathological and ultrasound imaging characteristics for predicting cervical lymph node metastasis in cN0 unilateral papillary thyroid microcarcinoma. Front Surg. (2021) 8:742328. doi: 10.3389/fsurg.2021.742328, PMID: 34926565 PMC8677692

[24] Khokhar MT Day KM Sangal RB Ahmedli NN Pisharodi LR Beland MD . Preoperative high-resolution ultrasound for the assessment of Malignant central compartment lymph nodes in papillary thyroid cancer. Thyroid. (2015) 25:1351–4. doi: 10.1089/thy.2015.0176, PMID: 26431908

[25] Zhang X Zhu J Ai X Dang M Huang P . An ultrasound-based nomogram for predicting central lymph node metastasis in papillary thyroid microcarcinoma. Med Ultrason. (2024) 26:369–75. doi: 10.11152/mu-4411, PMID: 39078991

[26] Kaliszewski K Zubkiewicz-Kucharska A Kiełb P Maksymowicz J Krawczyk A Krawiec O . Comparison of the prevalence of incidental and non-incidental papillary thyroid microcarcinoma during 2008-2016: a single-center experience. World J Surg Oncol. (2018) 16:202. doi: 10.1186/s12957-018-1501-8, PMID: 30305094 PMC6180613

[27] Wolff RF Moons KGM Riley RD Whiting PF Westwood M Collins GS . PROBAST: A tool to assess the risk of bias and applicability of prediction model studies. Ann Intern Med. (2019) 170:51–8. doi: 10.7326/m18-1376, PMID: 30596875

[28] Chen R Wang SF Zhou JC Sun F Wei WW Zhan SY . Introduction of the Prediction model Risk Of Bias ASsessment Tool: a tool to assess risk of bias and applicability of prediction model studies. Zhonghua Liu Xing Bing Xue Za Zhi. (2020) 41:776–81. doi: 10.3760/cma.j.cn112338-20190805-00580, PMID: 32447924

[29] Gao L Wen X Yue G Wang H Lu Z Wu B . The predictive value of a nomogram based on ultrasound radiomics, clinical factors, and enhanced ultrasound features for central lymph node metastasis in papillary thyroid microcarcinoma. Ultrason Imaging. (2025) 47:93–103. doi: 10.1177/01617346251313982, PMID: 39865963 PMC11783986

[30] Sun W Lan X Zhang H Dong W Wang Z He L . Risk factors for central lymph node metastasis in CN0 papillary thyroid carcinoma: A systematic review and meta-analysis. PloS One. (2015) 10:e0139021. doi: 10.1371/journal.pone.0139021, PMID: 26431346 PMC4592212

[31] Noel JE Thatipamala P Hung KS Chen J Shi RZ Orloff LA . Pre-operative antithyroid antibodies in differentiated thyroid cancer. Endocr Pract. (2021) 27:1114–8. doi: 10.1016/j.eprac.2021.06.014, PMID: 34217894

[32] Huang DM Zhi JT Zhang JM Zheng XQ Zhao JZ Wei SF . Correlations of serum TgAb and TPOAb and clinicopathological features of PTC in children and adolescents. Zhonghua Er Bi Yan Hou Tou Jing Wai Ke Za Zhi. (2022) 57:1418–25. doi: 10.3760/cma.j.cn115330-20220927-00581, PMID: 36707945

[33] Li X Zhang H Zhou Y Cheng R . Risk factors for central lymph node metastasis in the cervical region in papillary thyroid carcinoma: a retrospective study. World J Surg Oncol. (2021) 19:138. doi: 10.1186/s12957-021-02247-w, PMID: 33941214 PMC8091777

[34] Sun GH Qu N Hu JQ Shi RL Zhang TT Wen D . Risk for metastasis of lymph node between sternocleidomastoid and sternohyoid muscle in papillary thyroid cancer. Zhonghua Er Bi Yan Hou Tou Jing Wai Ke Za Zhi. (2017) 52:253–8. doi: 10.3760/cma.j.issn.1673-0860.2017.04.003, PMID: 28441800

[35] Wang Y Zheng J Hu X Chang Q Qiao Y Yao X . A retrospective study of papillary thyroid carcinoma: Hashimoto’s thyroiditis as a protective biomarker for lymph node metastasis. Eur J Surg Oncol. (2023) 49:560–7. doi: 10.1016/j.ejso.2022.11.014, PMID: 36404253

[36] Attia AS Hussein M Issa PP Elnahla A Farhoud A Magazine BM . Association of BRAF(V600E) mutation with the aggressive behavior of papillary thyroid microcarcinoma: A meta-analysis of 33 studies. Int J Mol Sci. (2022) 23:15626. doi: 10.3390/ijms232415626, PMID: 36555268 PMC9779545

[37] Virk RK Van Dyke AL Finkelstein A Prasad A Gibson J Hui P . BRAFV600E mutation in papillary thyroid microcarcinoma: a genotype-phenotype correlation. Mod Pathol. (2013) 26:62–70. doi: 10.1038/modpathol.2012.152, PMID: 22918165

