# Supplementary material for: Risk prediction model for cervical lymph node metastasis of papillary thyroid microcarcinoma: a systematic review and meta-analysis
Source: Front Endocrinol (Lausanne). 2025 Nov 18;16:1709773. doi: 10.3389/fendo.2025.1709773 (PMC12668969; doi:10.3389/fendo.2025.1709773)
Supplement: Supplementary file 1 [file SupplementaryFile1.doc]

**Appendix 1: Full Search Strategies**

**1. PubMed** ((("Carcinoma, Papillary"[Mesh]) OR "Papillary Thyroid Microcarcinoma" OR "PTMC") AND (("Lymphatic Metastasis"[Mesh]) OR "cervical lymph node metastasis" OR "CLNM") AND (("Prognosis"[Mesh]) OR "risk model" OR "prediction model" OR "nomogram" OR "risk score" OR "predictive factor"))

**2. EMbase** ('papillary thyroid carcinoma'/exp OR 'papillary thyroid microcarcinoma' OR 'ptmc') AND ('lymph node metastasis'/exp OR 'cervical lymph node metastasis' OR 'clnm') AND ('prognosis'/exp OR 'risk model' OR 'prediction model' OR 'nomogram' OR 'risk score' OR 'predictive factor')

**3. Web of Science** TS=("papillary thyroid microcarcinoma" OR "PTMC") AND TS=("lymph node metastasis" OR "CLNM") AND TS=("prediction model" OR "risk model" OR "nomogram" OR "prognosis")

**4. Cochrane Library** ([mh "Carcinoma, Papillary"] OR "Papillary Thyroid Microcarcinoma":ti,ab,kw OR "PTMC":ti,ab,kw) AND ([mh "Lymphatic Metastasis"] OR "cervical lymph node metastasis":ti,ab,kw OR "CLNM":ti,ab,kw) AND ([mh Prognosis] OR "prediction model":ti,ab,kw OR "risk model":ti,ab,kw OR "nomogram":ti,ab,kw)

**5. CNKI (China National Knowledge Infrastructure)** SU=('甲状腺微小乳头状癌' + 'PTMC') AND SU=('淋巴结转移' + 'CLNM') AND SU=('预测模型' + '风险模型' + '列线图')

**6. Wanfang Database** 主题:("甲状腺微小乳头状癌" OR "PTMC") AND 主题:("淋巴结转移" OR "CLNM") AND 主题:("预测模型" OR "风险模型" OR "列线图")

**7. Chinese Biomedical Literature Database (CBM)** "甲状腺微小乳头状癌"[常用词] AND "淋巴结转移"[常用词] AND ("预测模型"[常用词] OR "风险模型"[常用词] OR "列线图"[常用词])
